# A machine learning based model accurately predicts cellular response to electric fields in multiple cell types

**DOI:** 10.1038/s41598-022-13925-4

**Published:** 2022-06-15

**Authors:** Brett Sargent, Mohammad Jafari, Giovanny Marquez, Abijeet Singh Mehta, Yao-Hui Sun, Hsin-ya Yang, Kan Zhu, Roslyn Rivkah Isseroff, Min Zhao, Marcella Gomez

**Affiliations:** 1 Department of Applied Mathematics, University of California, Santa Cruz, CA 95064 USA; 2 Department of Earth and Space Sciences, Columbus State University, Columbus, GA 31907 USA; 3 Department of Dermatology, University of California, Davis, Sacramento, CA 95816 USA; 4 Department of Ophthalmology & Vision Science, University of California, Davis, Sacramento, CA 95817 USA

**Keywords:** Motility, Applied mathematics, Computational science, Scientific data, Computational models, Machine learning, Predictive medicine

## Abstract

Many cell types migrate in response to naturally generated electric fields. Furthermore, it has been suggested that the external application of an electric field may be used to intervene in and optimize natural processes such as wound healing. Precise cell guidance suitable for such optimization may rely on predictive models of cell migration, which do not generalize. Here, we present a machine learning model that can forecast directedness of cell migration given a timeseries of previous directedness and electric field values. This model is trained using time series galvanotaxis data of mammalian cranial neural crest cells obtained through time-lapse microscopy of cells cultured at 37 °C in a galvanotaxis chamber at ambient pressure. Next, we show that our modeling approach can be used for a variety of cell types and experimental conditions with very limited training data using transfer learning methods. We adapt the model to predict cell behavior for keratocytes (room temperature, ~ 18–20 °C) and keratinocytes (37 °C) under similar experimental conditions with a small dataset (~ 2–5 cells). Finally, this model can be used to perform in silico studies by simulating cell migration lines under time-varying and unseen electric fields. We demonstrate this by simulating feedback control on cell migration using a proportional–integral–derivative (PID) controller. This data-driven approach provides predictive models of cell migration that may be suitable for designing electric field based cellular control mechanisms for applications in precision medicine such as wound healing.

## Introduction

A great number of cell types, which have various functions, have been shown to migrate directionally in response to an electric field (EF) in a process known as galvanotaxis (also known as electrotaxis)^1–5^. Galvanotaxis may play a key role in many biological phenomena which are of significant medical interest, including wound healing^6–8^ embryo development^9,10^, and cancer metastasis^4,11^. It has been suggested that electric fields can be manipulated to guide these biological processes for purposes such as accelerating wound healing^12–14^ and suppressing metastasis^15^.

Many applications involving EF stimulation in tissue engineering apply either a constant EF stimulation, an EF stimulation with periodic changes in directionality at a fixed frequency, or combinations thereof^16^. The various forms of stimulation can each result in a distinct cellular response and may influence any number of features such as cell morphology, orientation, migration, and phenotype^16^. The mapping between EF stimulation and cellular response is complex and not yet understood in its entirety. Additionally, extended applications of electric fields can be detrimental to tissues. Thus, it is advantageous to design a dynamic EF signal to maximize the ratio of a cell’s responsiveness to current, while minimizing off-target effects and tissue exposure to the exogenous EF. Previous works have already presented experimental platforms that allow for programming of custom EF signals to direct cell migration^17–20^. With the aid of a predictive model, methods in control theory can be used to design optimal EF signals offline or on the fly for real-time feedback control of cell migration.

To this end, the development of accurate and robust predictive models of galvanotaxis is of paramount importance to efforts towards optimizing galvanotactic responses in order to control biological processes. While there have been many models of galvanotaxis^21–25^, there have been very few efforts to develop predictive models of single-cell galvanotactic dynamics^26^.

Cell migration is notoriously difficult to model because cells have complex nonlinear responses to numerous environmental cues^27^. In general, there are two standard modeling approaches which include mechanistic models and data-driven models. Mechanistic models of single-cell galvanotaxis are more informative about the driving processes behind motility induced by an EF, but usually lack predictive ability. The limited predictive models are capable of replicating ground truth cellular behavior as well as revealing the mechanisms by which EFs induce migration, but are unable to adapt predictions to different EF values without retraining^26^.

**Figure 1:**
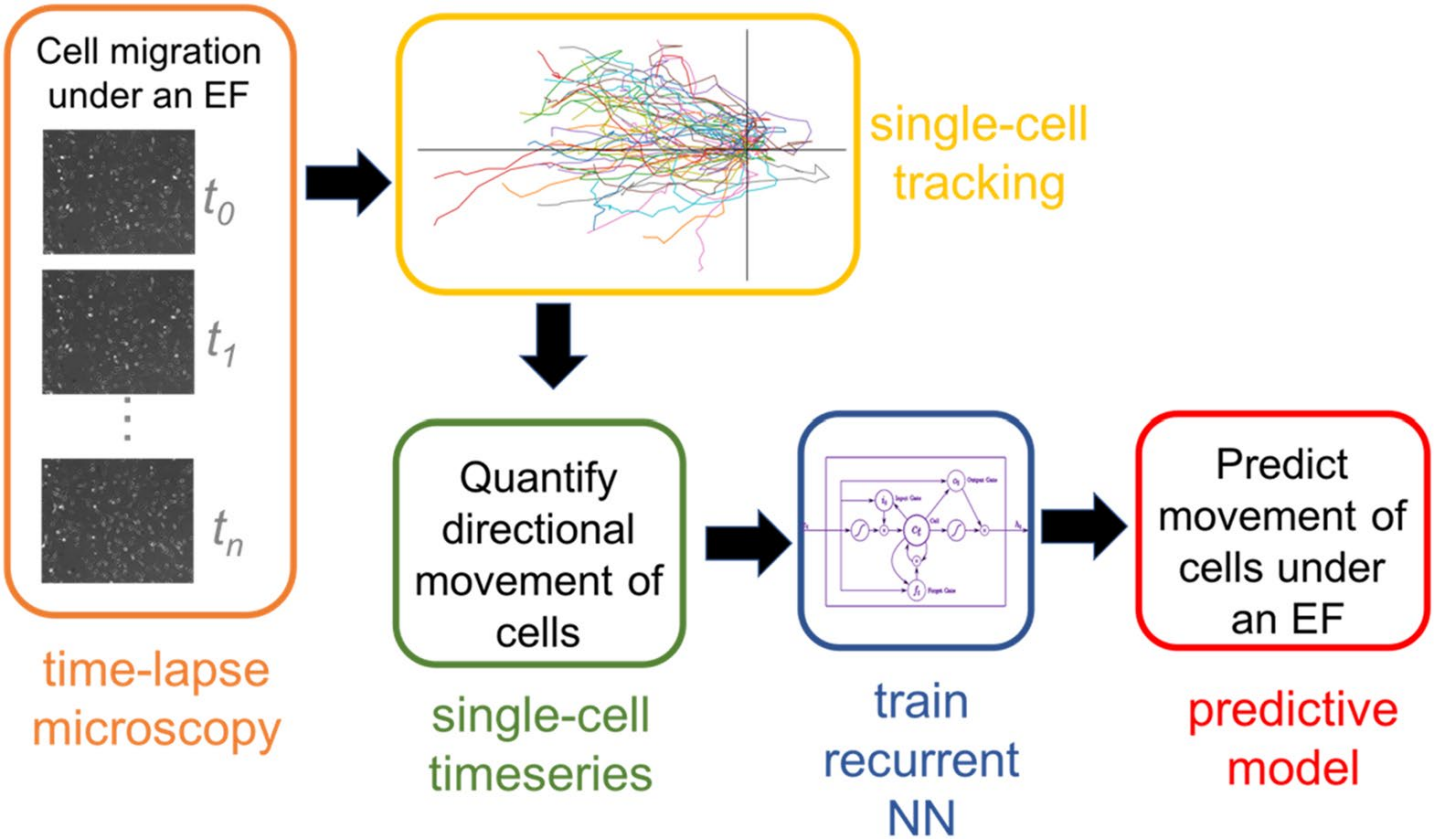
The image processing and cell directedness prediction pipeline. Time-lapse microscope images are used to manually track a number of cells. The tracking data is used to create timeseries of our two features (cell directedness and EF), which are used as inputs to our blackbox LSTM model to make predictions about the next directedness value.

In this paper, we propose a predictive deep learning-based approach to modeling EF-guided migration at the single-cell level. We note that this is the first machine learning based predictive model of galvanotaxis. Our proposed neural network-based model allows for prediction of migration directedness in a wide variety of experimental conditions, making it suitable for applications relevant to tissue engineering. Our models utilize a long short-term memory (LSTM) recurrent neural network architecture (see Fig. 1 for details), which has been shown to have great success in capturing temporal patterns for time series prediction tasks^28–30^. Our machine learning approach has several advantages over existing modeling approaches. Our model can take advantage of transfer learning methods to make accurate predictions on different cell types using limited training data. Our model takes as an input any time-varying or constant EF strength, and the predictive accuracy remains high when making predictions on EF values not encountered in training. The properties of our predictive model can serve two purposes. It can serve as an in silico experimental platform to save time and money on experiments while exploring the dynamic space of galvanotaxis. Second, it can be used to inform control algorithms to determine dynamic EF strategies to achieve desired cellular response. We demonstrate these two in the last two sections of the results through the generation of synthetic directedness data and by coupling it with a proportional–integral–derivative (PID) controller tasked with controlling cell migration patterns. The PID controller aims to control the average directedness of a population of cells. In summary, we present a versatile machine learning based model that can predict cellular response to changes in EF, while capturing stochastic cell migration patterns.

## Results

### Recurrent neural network models for galvanotaxis

Few machine learning models are suitable for timeseries data^31^. A popular category of ML models able to learn dynamical systems are known as recurrent neural networks (RNNs). Recurrent neural networks have a property such that the output of the model is fed back into the model as an input. If a dynamical system can be represented by an ODE, then a RNN can approximate the governing equations. The next question is whether galvanotaxis can be accurately modeled by an ODE given the stochastic characteristics of cell migration. RNNs are deterministic models but they can approximate stochastic behavior by converging to a chaotic model^32^. In this work, we show that such a chaotic model provides a good approximation to variability seen in cell migration.

**Figure 2:**
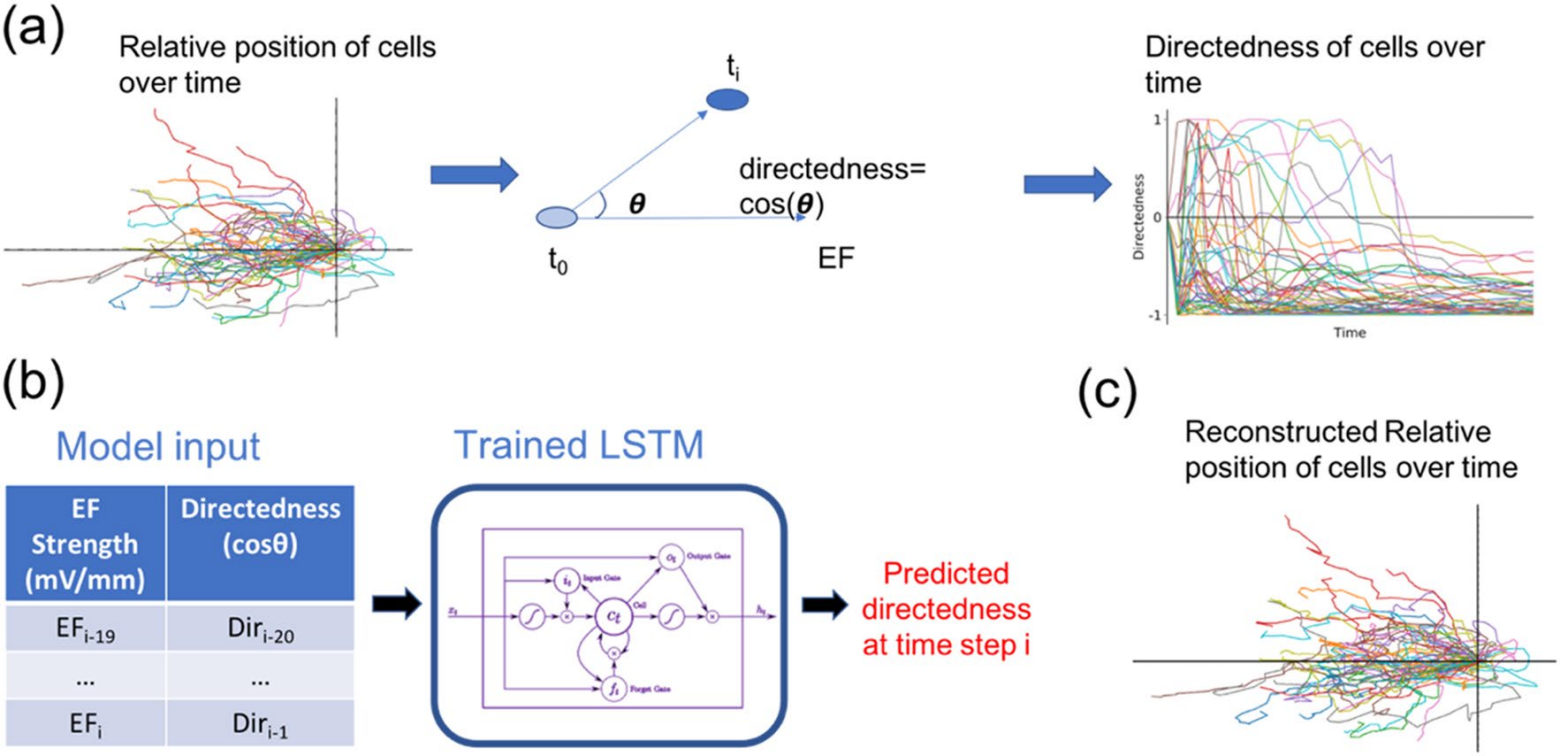
(**a**) Quantifying directional movement of cells by directedness. (**b**) Trained LSTM model takes in directedness and EF values from the past 20 times steps and outputs directedness at current time step. (**c**) Reconstruction of single-cell trajectories from directedness assuming constant cell speed.

We use a long short-term memory (LSTM) recurrent neural network (see Supplementary Fig. S1 for more details) to predict the direction of cell migration based on previous measured angles of migration and the current strength of the electric field (see Fig. 2 for details, and Table S1 for sample data). This is also referred to as a one-step ahead prediction. It has been shown that cell movement can be completely described mathematically using the speed and the angle of migration^25^. Furthermore, the speed is independent of the EF. Thus, our model only needs to consider the angle of migration. Here, the angle of migration is referred to as directedness and defined as the cosine of the angle between the electric field and the straight line which connects the centroid of the cell from its initial to current location. We note that simulations of cell trajectories can be reconstructed using directedness values. Figure 2c shows a reconstruction of single cell trajectories from computed directedness assuming constant speed. LSTM models have feedback connections and are designed to explicitly avoid the vanishing gradient problem, meaning that they can process entire sequences of timeseries data^33^. LSTM networks are advantageous over other recurrent networks since they are relatively insensitive to the duration of time delays^34^. These advantages make LSTM models desirable for understanding complex systems, and LSTM models have had success capturing the behavior of noisy dynamical systems^35,36^.

LSTM networks, like all neural networks, are trained by iteratively updating the internal weights of a network, which are usually randomly initialized, to minimize the loss function on the training set. For multilayer neural networks, including RNNs, this loss function is nonconvex in general. Thus, the weights found in the training stage are not guaranteed to represent a global minimum of the loss function, and the exact local minimum found is dependent on the initial weights. We use a fivefold cross-validation to evaluate the dependence of the model performance on the training set. To ensure that results are not dependent on any one set of initial weights, we train 50 randomly initialized models with identical architecture on the same training set. We then evaluate the performance of all 50 models so that the results reflect the overall performance of this modeling approach. All of our presented results show predictions of all models on every cell to demonstrate that these results are not dependent on any one random weight initialization.

### Recurrent NNs can predict the directedness of EF-induced cell migration at the single cell level

We first demonstrate the ability of the LSTM model to capture cell migration patterns under an electric field by predicting cell directedness one step ahead, given measured cell directedness at previous time points. We first train and test the model on a collection of timeseries Cranial Neural Crest Cell (CNCC) data^1^ capturing single cell migration under a set of EFs: 0 mV/mm, 15 mV/mm, 30 mV/mm, 50 mV/mm, 75 mV/mm, 100 mV/mm, and 200 mV/mm (see “Materials and methods”). To evaluate the model’s accuracy, we consider the distribution of root mean squared error (RMSE) values for single cell trajectories over a population of cells when comparing predicted directedness at each time step to the measured ground truth.

**Figure 3:**
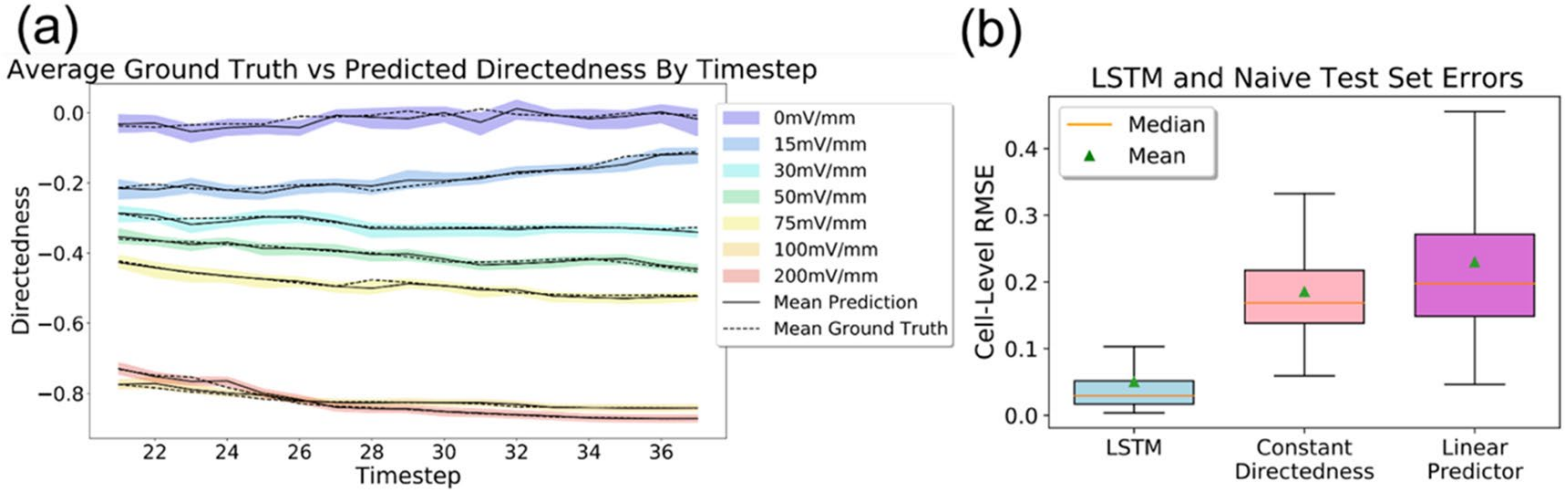
(**a**) Average predicted directedness and distribution across 50 models compared to ground truth measurements at each EF. A timestep unit is 5 min (the interval at which images were taken). The first 19 time steps are used to initiate predictions. (**b**) Comparison of LSTM model to naïve predictors. Root mean square error (RMSE) values computed based on predicted directedness and measured directedness. The boxes represent the middle 50% of error values and the whiskers extend to the minimum and maximum error values.

Figure 3 shows the results of predicting single-cell behavior for all EFs. In particular, the median values and their distribution across the 50 models are plotted against time. See Table S2 for the distributions of RMSE values when predicting on the training, validation, and test sets. The center and spread of the RMSE distributions for the training and test sets are comparable, implying that the model is not overfit. This is further supported by model simulations in a later section. See table S3 for cross-validation results using different combinations of standardized features. Standardization did not improve performance and was therefore not used further.

Additionally, to demonstrate that the predictions are indeed informed we compare the results to those of two naïve predictors (see Fig. 3). The first of these predictors, which we will call the “constant directedness” model for this discussion, makes a naïve assumption that directedness will remain constant from one timestep to the next. So, the directedness prediction made by this naïve model is just the previous directedness value. The second naïve predictor, which we refer to as the “linear” predictor, makes a linear extrapolation using the previous two directedness values. That is, the rate of change of directedness between the previous two timesteps is assumed to remain constant between the previous timestep and the next timestep. Figure 3 shows the error distributions of the naïve predictors alongside that of our base model, the LSTM. See Table S4 for the median RMSE values and corresponding IQR values. It’s interesting to note that simply assuming no change in directedness leads to a better approximation than a linear extrapolation. Thus, prediction of directedness is non-trivial even under a constant EF and it is clear that cell migration is driven by underlying dynamics.

### Recurrent NNs can predict the directedness of EF-induced cell migration at unseen EFs

**Figure 4:**
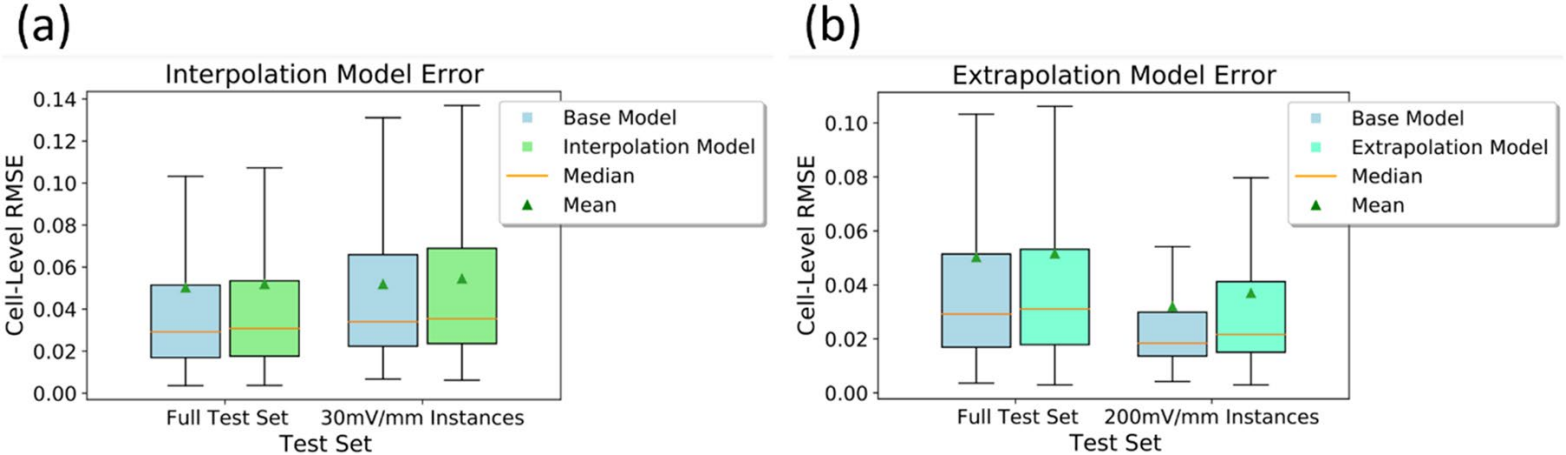
(**a**) Distributions of cell-level test set RMSE values of the base model and a model with identical architecture which was trained with a modified training set from which the 30 mV/mm instances were removed. Error distributions shown for both the complete testing set and for the 30 mV/mm test instances. (**b**) Distributions of cell-level test set RMSE values of the base model and a model with identical architecture which was trained with a modified training set from which the 200 mV/mm instances were removed. Error distributions shown for both the complete testing set and for the 200 mV/mm test instances. The boxes represent the middle 50% of error values and the whiskers extend to the minimum and maximum error values.

To understand the generalizability of the model with respect to the EF strength, we use the same modelling framework to both interpolate and extrapolate to EF strengths that were not seen in the training set. For interpolation, we remove all instances of cells in an intermediate EF, 30 mV/mm, from the training set and train a new model with identical architecture as before. For extrapolation, we follow a similar approach except we remove all instances of cells in an extreme EF, 200 mV/mm from the training set. The model is then tested on the complete data including unseen EFs. We also highlight the performance exclusively on cell trajectories under EFs omitted during training. First, we evaluate the ability of the model to interpolate to unseen EF strengths by considering the performance of the model trained without 30 mV/mm instances on all cells in the test set (see Fig. 4), as well as exclusively on the 30 mV/mm test instances. On both the full test set and the 30 mV/mm test set instances, the median RMSE of the interpolation model is only moderately higher than the base model (~ 5%). Additionally, the performance of the interpolation model on the 30 mV/mm instances alone is comparable to the performance on the full training set (see Table S5), meaning that the model interpolates well to unseen EF strengths.

For extrapolation, we compare the base model to a model trained without any 200 mV/mm instances (see Fig. 4). The median RMSE of this extrapolation model, when evaluated on the full test set is ~ 6.5% higher than the base model trained on the full training set. When evaluating this model specifically on the 200 mV/mm, the median RMSE is ~ 17.4% higher than that of the base model. See Table S5 for median RMSE and corresponding IQR distribution values. We note that the base model predicts directedness exceptionally well at 200 mV/mm when compared to its performance on the full test set. This implies cell migration is more predictive at this higher EF and removing this data when training the extrapolation model results in a noticeably increase in error across the full test set.

For both the interpolation and extrapolation tests, the model trained on the limited training set performs worse than our original model overall. This is to be expected given an overall smaller dataset. However, the acceptable performance exclusively on omitted EFs demonstrates the ability of our model to interpolate and extrapolate with respect to EF values, with relatively better performance on the interpolation task than on the extrapolation task.

### Transfer learning allows for high prediction accuracy when minimal data is available

**Figure 5:**
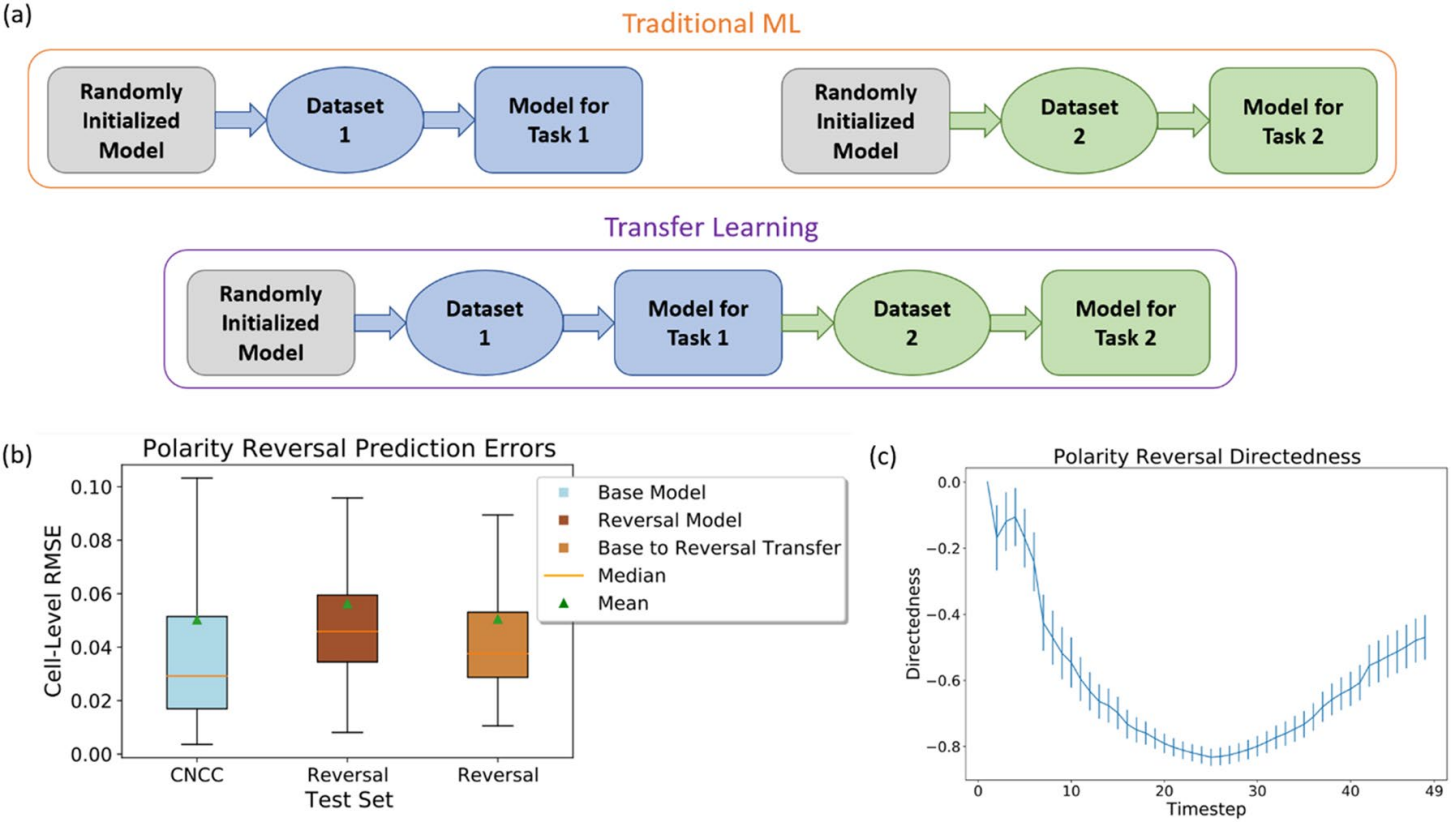
(**a**) Diagram comparing the traditional machine learning training approach, which involves training a separate randomly initialized model for each learning task, with the transfer learning approach, which involves training a model for one task and then retraining that model on another dataset to perform a related task. (**b**) Distributions of cell-level RMSE values for the base model, a model training only on the reversal dataset, and a model which uses transfer learning to retrain the base model for the polarity reversal task. The boxes represent the middle 50% of error values and the whiskers extend to the minimum and maximum error values. (**c**) Plot of average directedness over time for the polarity reversal dataset, with error bars representing standard error of the mean for each timestep to illustrate the spread of directedness values at each step.

Transfer learning is the method of using a model’s knowledge about one learning problem (called the source domain) to improve the performance on a second, related learning problem (called the target domain)^37–41^. While the traditional approach to machine learning requires learning a separate randomly initialized model from each domain’s training set to converge on a model specific to the task it was trained on, transfer learning involves learning a model for the source domain and then using that trained model as the starting point for learning the model for the target domain (see Fig. 5a). Transfer learning allows for target domain instances to be in a different feature space and have a different distribution than the instances in the source domain, which allows for relatively high performance when target domain data is too limited to allow for such performance were the model to be trained from a random initialization^40,41^. Because galvanotaxis experiments and manual cell tracking can be both expensive and time-consuming, galvanotaxis tracking datasets for some cell types may be limited in both the number of cells tracked and the variety of EF conditions in which experiments are conducted. Thus, transfer learning may be a pivotal tool in developing accurate models for cell types and experimental conditions for which data is limited. Here, we evaluate the effects of transfer learning on extending our constant EF CNCC model to different cell types and to a time-varying EF.

First, we consider transfer learning methods for making predictions about cells in time-varying EFs using the model which we trained on constant EFs. We evaluate the ability of the model to capture CNCC galvanotaxis dynamics in an experiment in which the polarity of a 200 mV/mm EF is reversed halfway through the experiment (see Fig. 5). We compare the performance of a “reversal model”, trained only on the polarity reversal data, and a “transfer learning model”, which retrains the base model on the polarity reversal data. Once again, we use the base model predictions on the constant-EF test set as a performance benchmark.

The median RMSE of the reversal model is ~ 57.2% higher than the benchmark performance of the base model on the original test set. The transfer learning model provides an improvement of ~ 18.1% over the reversal model. The transfer learning model’s median test set RMSE is ~ 28.8% higher than the benchmark model’s median RMSE, which can likely be attributed to both the limited polarity reversal training data, as well as the increased complexity of dynamics in the time-varying EF setting. See Table S6 for median RMSE values. Despite the inability of the model to reach benchmark performance on this task (note that only one dataset with dynamic EF was available), we have demonstrated that transfer learning methods are effective at improving model performance for cells in time-varying EFs over models trained only in those settings.

Next, we evaluate the effectiveness of transfer learning methods for extending our method to cell types with limited galvanotaxis tracking data. We consider the application of our CNCC model to both fish keratocytes and human keratinocytes. Both of these cell types have been shown to migrate towards the cathode of an electric field^2,3^, while CNCC migrate towards the anode^1^. Thus, the model must learn to predict galvanotactic behavior which differs significantly from the behavior of CNCC. Using limited training sets for both target cell types, we compare the performance of a model that uses the same architecture as our original model but has been trained only on the target data with our CNCC model, which we have retrained using the same target data using transfer learning methods. Again, we use the performance of the CNCC model as a benchmark, as our goal is that these models, once transfer learning methods have been applied, can have target data test performance similar to the benchmark test performance on the CNCC data.

We have one keratocyte dataset and two keratinocyte datasets. The keratocyte data contains tracking timeseries for 0 mV/mm, 50 mV/mm, 100 mV/mm, 200 mV/mm, and 400 mV/mm electric fields. All keratinocyte cells are tracked in 100 mV/mm EFs. The keratocyte training set contains tracking data for two cells from each available EF strength and the two keratinocyte training sets each contain tracking data for two cells total. For keratocytes, images are taken, and cell positions are recorded every 30 s. The first keratinocyte dataset records positions at one-minute intervals, while the second keratinocyte dataset has positions recorded at ten-minute intervals. Thus, this task evaluates not only the ability of the model to transfer knowledge to other cell types, but also the ability of the model to adjust to different timescales.

**Figure 6:**
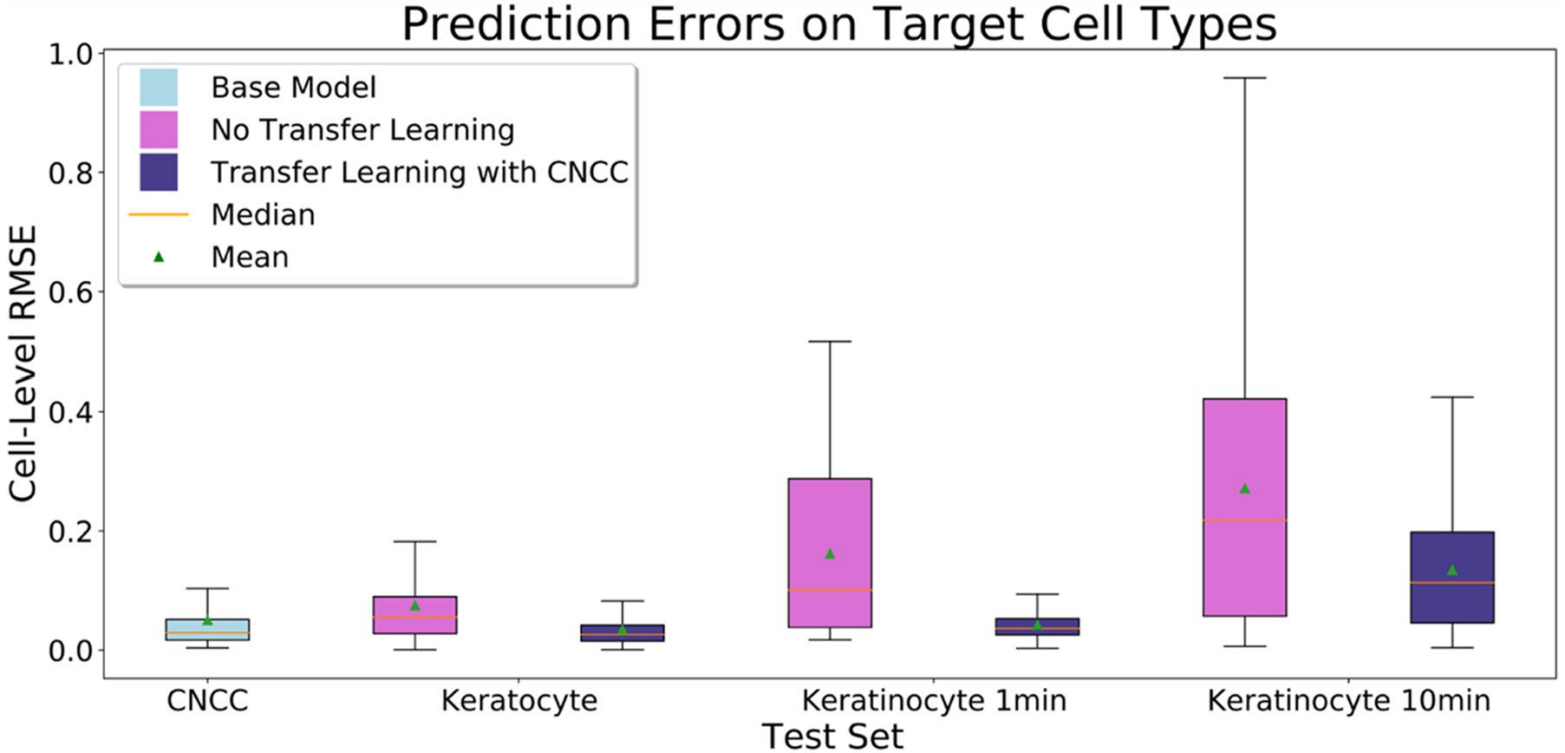
Distributions of RMSE values for the base model on the CNCC test set (benchmark), the keratocyte model on the keratocyte test set, and the transfer learning model on the keratocyte test set. Distributions of RMSE values for the base model on the CNCC test set (benchmark), the target cell models without transfer learning on the target cell test sets, and the target cell models which used transfer learning with the CNCC source domain on the target cell test sets. The boxes represent the middle 50% of error values and the whiskers extend to the minimum and maximum error values.

Our keratocyte model, trained only on the keratocyte data, has a median RMSE of 0.0554 on the test set, which is ~ 189.7% higher than the median RMSE of the benchmark model performance on the CNCC test set. For transfer learning, we take the CNCC model and retrain the weights on the keratocyte training set, resulting in a median RMSE of 0.0260 on the keratocyte test set, which is ~ 53.1% lower than the keratocyte model which did not use transfer learning and ~ 11% lower than the benchmark performance on the CNCC dataset. So, the model trained only on our limited keratocyte data has much higher median RMSE than the benchmark, while the transfer learning model achieves lower median error than the benchmark (see Fig. 6).

The first keratinocyte model, the model trained only on the one-minute interval keratinocyte dataset, has a median RMSE of 0.1006, ~ 244.5% higher than the benchmark median RMSE. The transfer learning model, in which the base model was retrained using the same keratinocyte training set, has a test set median RMSE of 0.0362, which is ~ 64% lower than the median error of the model only trained on the keratinocytes, and is just ~ 24% higher than the median RMSE of the benchmark CNCC model. The spread of the error distribution was also much lower for the transfer learning model than for the keratinocyte-only model, with a RMSE IQR of 0.0274 for the transfer learning model and 0.2502 for the keratinocyte-only model (see Fig. 6).

The median RMSE of the model trained only on the ten-minute interval keratinocyte dataset when predicting on the test set is 0.2176, which is ~ 645.2% higher than the benchmark median RMSE. After retraining the CNCC model on the ten-minute interval keratinocyte training set, the resulting model has a median RMSE of 0.1134, which is ~ 288.4% higher than the benchmark model, but ~ 47.9% lower than the keratinocyte model that did not use transfer learning. Once again, the spread is significantly lower in the model that used transfer learning, with a RMSE IQR of 0.1059 in the transfer learning model and 0.2383 in the keratinocyte-only model (see Fig. 6). The unusually large increase in error overall may be due to the significantly increased sampling time from 5 to 10 min.

While transfer learning in these cases did not always lead to performance comparable to the benchmark, both the median and IQR of RMSE distributions were much lower for the transfer learning model than for the model trained only on the target cell type in all cases. We have shown that transfer learning can be an effective approach for developing predictive models about cell types for which available data is limited, even when the source cell data differs from target cell data in significant ways, such as the time interval between observations and anodal- versus cathodal-directed migration.

### NN-based models can be used for in silico studies

In recent years, the massive increase in the quantity of available data has led to much attention being paid to in silico biological studies, which are studies performed on computers using mathematical modeling and simulations^42–45^. The advantages of in silico studies include estimating hidden system parameters that are experimentally inaccessible^46^, optimizing the timeline of experimental procedures and product development^47,48^, reducing the need for animal and human trials^48^, and lowering experimental costs^47–49^. In this section, we demonstrate that the recurrent neural network-based model that we have developed can be used for in silico galvanotaxis assays with arbitrary and time-varying EFs.

We simulate cell migration experiments by designing an EF timeseries and using some initial ground truth data to begin making predictions. In this way, we can generate timeseries of synthetic galvanotaxis tracking data using arbitrary EF values, which may vary in time. We compare the distributions of synthetic directedness values with those from the ground truth data to evaluate the ability of the model to capture the long-term effects of EFs on CNCC.

The specific comparison we consider is between the distributions of the directedness values at the end of the experiments. Our simulations use 20 timesteps of initial ground truth data to begin making predictions and each CNCC is tracked in a constant EF for 37 timesteps after the initial image. Thus, we are comparing how the ground truth directedness distribution evolves in the final 17 timesteps with the evolution predicted by the simulation in the same time period. The ground truth data we consider are the 350 cells in the test set. These cells are used for the initial lookback to begin the simulations and for the comparison with ground truth final directedness values.

**Figure 7:**
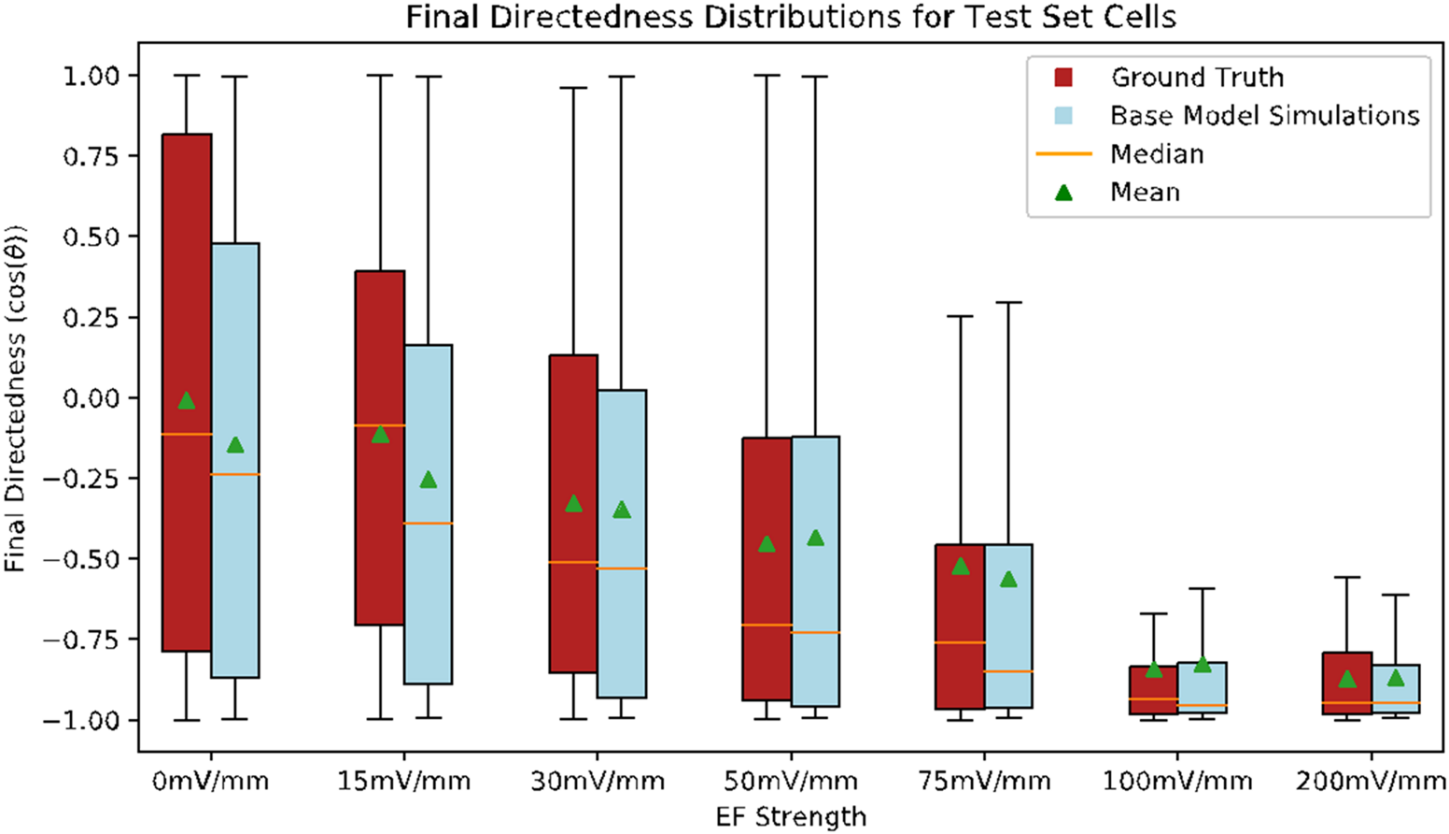
Distributions of final directedness values of cells in both ground truth data and synthetic data generated by simulations. The boxes represent the middle 50% of cell directedness values and the whiskers extend to the minimum and maximum directedness values for each distribution. For simulations, these distributions are over 50 trained models to ensure that these results are not dependent on the random initialization of any one model; see “Materials and methods” subsection “Recurrent model architecture” for more details. See Table S7 for mean and median values of final directedness values for both ground truth and synthetic data.

**Figure 8:**
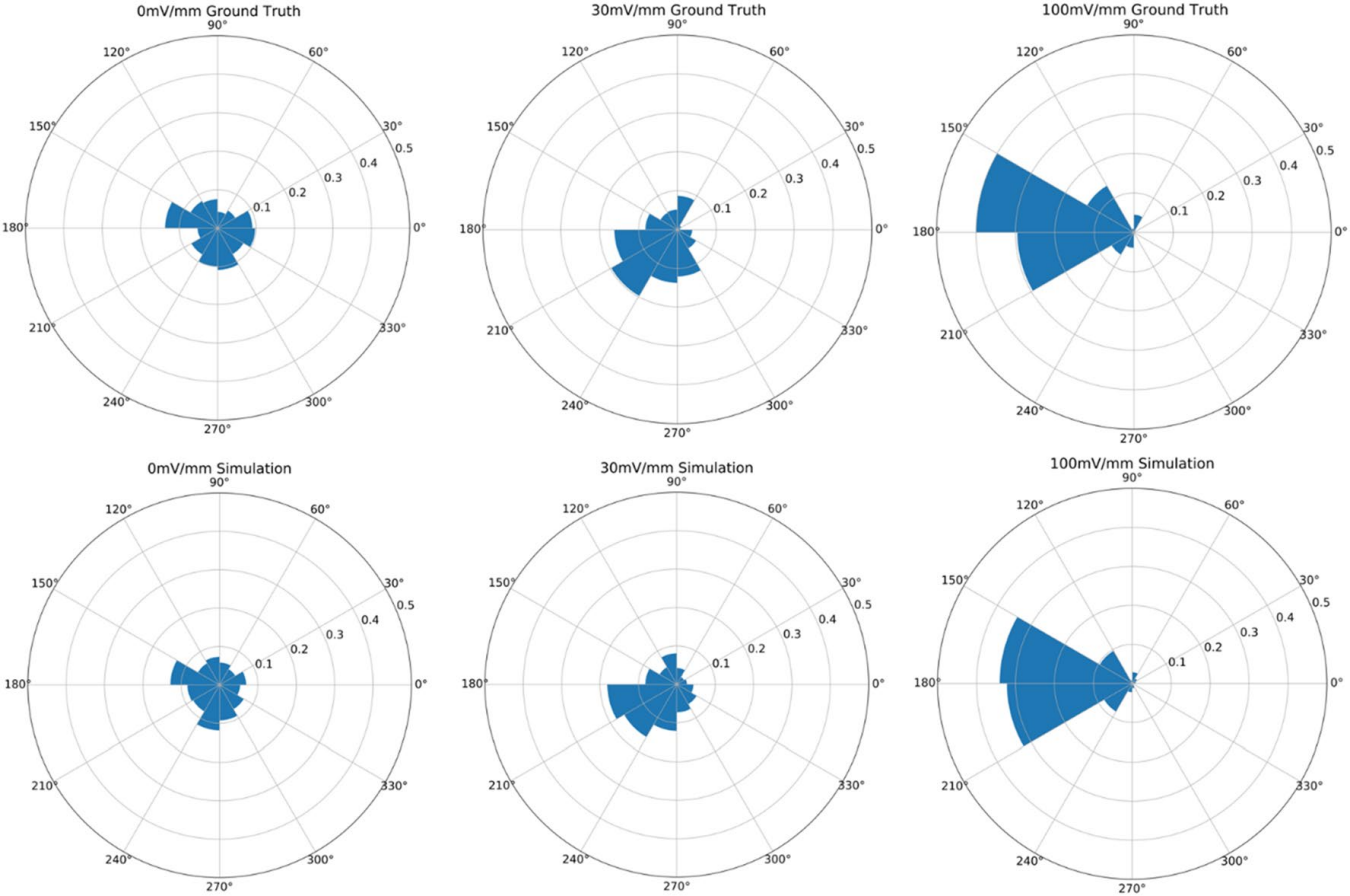
Directional plots for cell migration at various EF for ground truth measurements and in silico simulations with added noise on computed directedness at each time step.

To determine the ability of our model to replicate the effects of an EF on cell motility in silico, we compare the distribution of final directedness values of the in silico synthetic data against the ground truth data across all the EF values in the CNCC dataset (see Fig. 7). In Fig. 8, we show the circular distribution of the directional data for a subset of the EFs. We compare the directedness values by EF to evaluate whether the model has learned the effects of various EF values on the cells. If the distributions of EF-level predicted directedness values are similar to those of the EF-level ground truth directedness values, we can conclude that the in silico studies capture the general migration behaviors of the CNCCs.

The means and medians of final directedness values computed by the simulations are closely correlated with the ground truth. The correlation coefficient between the means is R = 0.9906 and between the medians is R = 0.9721. In general, the distributions of simulated and ground truth final directedness values get closer as EF strength increases and cell behavior becomes more predictable.

Specifically, there is a significant drop in the differences between both means and medians at 30 mV/mm and higher, compared to 0 mV/mm and 15 mV/mm simulations. The threshold of response of CNCC to electric fields has been identified as being in the range between 15 mV/mm and 30 mV/mm^1^, so we expect that the simulations will more closely reflect the ground truth in EF strengths above that threshold due to the largely stochastic behavior of cells below the threshold.

To get a better sense of the distribution of directionality across cells we also present cell motility in polar coordinates, where the angle represents the direction of motion (with 30 degree bin widths) and the radius represents the proportion of cells moving in a given direction. To create these rose plots using our synthetic data, we must map the directedness values generated by the model to (x, y) positions. These positions cannot be recovered exactly from the directedness values alone, so we approximate them using previous positions, as assumption of cell speed, and the previous heading of the cell (see “Materials and methods” for more details). This approximation was shown to provide fairly accurate results in Fig. 2. We note that the LSTM model is ultimately a deterministic model and so directedness can converge to deterministic equilibrium point over long simulations. Figure 8 shows comparison of rose plots for experimental data and data generated by the LSTM model in silico.

### In silico demonstration of feedback control on cell migration

**Figure 9:**
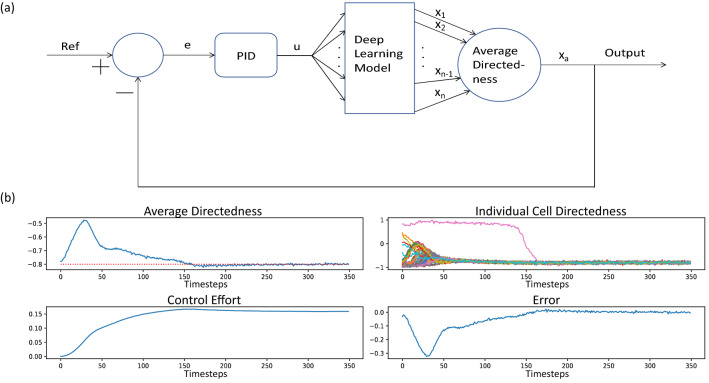
(**a**) The top figure depicts the closed loop design. A reference value/trajectory is picked. The error is evaluated and fed to the PID. The PID uses this value to determine the appropriate EF in order to guide the average directedness towards the reference. This EF is applied to the model of cell migration. (**b**) (Bottom upper left) The average directedness of all the cells (the dashed red line indicates the reference value). (Bottom upper right) The directedness of individual cells. (Bottom lower left) The voltage being applied throughout the simulation to get the appropriate response. (Bottom lower right) The error of the average directedness in relation to the reference value.

In this section we demonstrate the utility of the model to design and simulate a feedback algorithm to control cell directedness by real-time regulation of the EF. We present an in silico study, applying a PID controller to evaluate the EF necessary to keep the average cell directedness at a certain reference value. Multiple cells are simulated using one of the 50 LSTM models. The cells’ directedness is averaged and used as the measured state for output feedback control. Figure 9a shows the details of the closed loop control system and simulation results. The reference directedness was set to − 0.8. 5% gaussian noise was added to the output of the model to maintain stochasticity. As seen in Fig. 9b, individual cells achieve the desired directedness by applying the appropriate voltages derived from the PID. This implies a possibility to be able to control cell directedness through feedback control in an in vitro setting. We note that the PID was carefully tuned for the particular model chosen. More work remains to be done in the development of feedback control algorithms with guaranteed convergence under varying experimental conditions affecting measured response.

## Discussion

Galvanotaxis has been observed in many cell types and plays key roles in processes such as wound healing and cancer metastasis. Here, we have presented a recurrent deep learning model which can capture the single-cell directedness dynamics of cranial neural crest cells. We have demonstrated that our deep learning model can make accurate predictions in constant-strength electric field settings even in EF strengths that were not seen by the model during the training stage. We have also shown that our model can generate simulations of galvanotactic behavior like those we see in our experimental datasets. The value of these simulations is twofold. First, the similarities between the synthetic data generated by our model and the ground truth tracking data from real experiments illustrate that the predictions made by our model in response to various EF strengths are in line with how we expect real cells to behave given our dataset and literature on galvanotaxis tracking data. Second, these simulations demonstrate the potential for our recurrent models to perform in silico galvanotaxis migration experiments. Such experiments may be used in place of physical experiments, which would allow for reducing experimental costs and rapidly designing and implementing new experimental setups.

Another key strength of this approach is the ability of our trained model to make reasonable predictions on other cell types after retraining on a very limited sample of the target cell data, even when the target cells exhibit different electrotactic behavior from the original cell type. By training our model on a single rich dataset, we can extend the model to a variety of cell types for which similarly rich datasets are unavailable. We show successful results in transfer learning with as few as 2 cells from the new dataset. As galvanotaxis experiments and tracking procedures can be costly and time-consuming, the ability of our model to converge in the retraining stage for small datasets may allow for predictive models to be created with low experimental costs.

There have been some previous models of galvanotaxis^21^, and recently mathematical modeling has been used to quantify motility at a single-cell level^26^. However, to our knowledge, this work is the first to present a predictive deep learning model that can predict single-cell behavior one time-step ahead, and the first model of any type to consider the response of cells to time-varying EF strengths. Further, the machine learning approach allows us to easily transfer learned parameters from one cell type to aid in the modeling of other cell types. The ability of the model to both interpolate and extrapolate to new EF strengths and its performance on the polarity switch experiment, along with the generation of qualitatively reasonable synthetic data, suggest that our model can be used to inform effective time-varying EF signals in applications of control for precision medicine. One of the potential applications of galvanotaxis models is informing controller-based intervention in wound healing and metastasis processes, and it is necessary for such models to be able to adapt to changing EFs without retraining. The effects of galvanotaxis vary by EF strength^1,3^, so a control policy capable of modulating EF strength may allow for more precise control of cells. Higher precision in directing cell migration would allow for more efficiency and consistency when influencing galvanotaxis, allowing for more exact optimization of the biological processes we wish to guide.

While there are some promising studies of galvanotaxis in vivo^19,50^, most galvanotaxis studies are done in vitro^16^ due to the difficulty of imaging cell migration in vivo. Such experimental designs are standardized, it would be advantageous to standardize and automate the modeling of the effects of galvanotaxis. The development of general data-driven galvanotaxis modeling techniques would allow for automated analysis of arbitrary galvanotaxis experiments using standard cell tracking data. Such automated modeling could open the door to deeper understanding of the dynamics of galvanotaxis. For example, this work can be expanded to capture collective cell behavior by incorporating information on spatial distribution of cells. In this work, cell density is kept small to ensure that cells do not touch. We expect differences between single cell migration in the EF and migration of sheets of cells joined by intercellular connections^51–54^. Modeling collective behavior would be important to understanding dynamics such as in epithelialization, where keratinocytes migrate to cover the wound bed^55^.

The consideration of the effects of intercellular connections is but one of the potential extensions of our work that can be systematically explored with a standardized modeling approach. Other extensions that may improve the interpretability or predictive power of such modeling include the explicit inclusion of random variables in the model to avoid the long-term steady-state behavior we observed with our deterministic approach, as well as experimentation with other features that may act as good short-term predictors to allow models to use a shorter lookback with similar performance. We have not explored these potential improvements in this work but mention them here to acknowledge the open problems that may be investigated with a standardized machine learning driven model of galvanotactic behavior such as the one we have presented.

## Materials and methods

### Galvanotaxis assay

The CNCCs data used in this study are from O9-1 ​cell lines, which is an extended culture of primary CNCCs isolated from E8.5 mouse embryo (courtesy of Dr. Xinli Zhang, UCLA School of Dentistry). The cells were loaded into the electrotactic chamber (pre-coated with 1:50 diluted Matrigel) and incubated for 4 h to allow for attachment. Agar/saline bridges were placed into the chamber channels for application of DC electric field^1^.

To generate primary fish keratocytes, scales were removed from the flanks of black skirt tetra *Gymnocorymbus ternetzi* and allowed to adhere and grow onto the bottom of a culture dish at room temperature in Leibovitz’s L-15 media (Gibco BRL), supplemented with 14.2 mM HEPES pH 7.4, 10% Fetal Bovine Serum (Invitrogen), and 1% antibiotic–antimycotic (Gibco BRL). Sheets of keratocytes that migrate off the scale after 24–48 h were dissociated, seeded in another tissue culture dish, and incubated at room temperature for 1–3 h to allow for attachment. The galvanotaxis experiments were performed using custom-made galvanotaxis chambers^3^.

Isolation of human keratinocytes was performed on the discarded foreskin of elective circumcision from de-identified neonatal male donors at UC Davis hospital in Sacramento, CA (PMID: 19143471). Foreskin sample collecting was done under a protocol approved by the UC Davis Institutional Review Board (IRB) Administration. NHK between passage 2–5 were plated on collagen coated galvanotaxis chambers at 6–8 × 10^4^ cell/ml for 2 h to allow the cells to attach and migrate. A 100 mV/mm DC electric field (EF), comparable to the physiological range at the wound field, was applied to the chambers for galvanotaxis^2^.

As for temperature—Keratinocytes and neural crest cells were in 37 C, while keratocytes were cultured at room temperature ~ 18–20 °C. All assays were at atmospheric pressure.

### Microscopic imaging and cell tracking

For the CNCC, images were taken at 5-min intervals for 180 min using a Zeiss Axio Observer Z1 inverted microscope with a QImaging Retiga R6 CCD camera. The cells were tracked manually using ImageJ (National Institutes of Health) software^1^.

Keratocytes were maintained in the culture medium at room temperature. Time-lapse images were recorded on a Zeiss Axiovert 40 with a motorized stage, a 10 × lens, and a Hamamatsu C4742-95 CCD digital camera. Images were taken at 30-s intervals for up to two hours. Time-lapse images were processed with ImageJ software (http://rsbweb.nih.gov/ij). Cells were tracked by using the MtrackJ tool. Migration speed and directionality were defined and calculated as previously described^3^.

For the keratinocyte data, time-lapse Images (time interval of 1 min for 60 min for the first keratinocyte experiment and time interval of 10 min for 410 min for the second dataset) were acquired on a Nikon TE-2000 microscope with a motorized stage, an environmental chamber to maintain at 37 °C, a 20 × Nikon Plan Fluo objective, a Retiga EX camera (Qimaging, Canada), and the Volocity imaging software (PerkinElmer, Waltham, MA). Cell tracking was manually performed with OpenLab software and the cell migration rate and directionality of galvanotaxis were calculated^2^.

### Dataset creation

Training and testing datasets were constructed using the cell position time series. For each cell position, the directedness is measured as the cosine of the angle between the EF and the straight line connecting the cell’s current position with its starting position. While we typically discuss EF strengths as mV/mm because that is the SI unit for electric fields, the EF strength input to the model is in V/mm to put the order of EF inputs on a similar order as the directedness values. That is, the only pre-processing done on the data was a rescaling on the input data. No other processing was applied. At each timestep, our model uses the directedness and the EF strength. Because the LSTM has a 20 timestep lookback, each instance contains the 20 previous directedness values, the 19 previous EF values, and the next EF value (as features) along with the next directedness value (as the prediction target). There was no missing data. Every cell that appeared in every time frame through the course of the experiment was included. There were no missing time points.

### Recurrent model architecture

Our model was constructed and trained using Keras running on a TensorFlow backend. The model has a lookback of 20 timesteps and the data contains the directedness and EF strength at each timestep, so the input layer accepts matrices of shape (20, 2).

Our primary model contains a single LSTM layer with 80 units which uses a hyperbolic tangent activation function and a sigmoid activation function for the recurrent step. The LSTM layer is densely connected to a single output unit using the hyperbolic tangent activation function.

The loss was measured as the square of the error and backpropagated through the network for each instance; that is, the batch size is 1 and the loss function is the squared error. To minimize prediction error, the loss was backpropagated through the network and weights were updated using the Adam optimizer with a learning rate of 0.001.

To ensure that the performance of our model is not an artifact of the random initialization of the weights, we train 50 versions of the model with identical architecture and training procedure. The weights of each model are initialized randomly, and we consider the distributions of predictions over all 50 models, thus avoiding a reliance of our results on any initialization. When presenting results in the form of distributions of cell-level RMSE values, we calculate the RMSE for all predictions on a single cell for an individual model and report all RMSE values from all models over all cells in the test set.

We note that different look back sizes were considered and a lookback of 20 provided the best performance. Additionally, more complex architectures were considered but these models also did not perform as well. We presume this is due to the size of the data.

### Implementation of transfer learning methods

For the weight initialization transfer learning method which we use for predicting both, we begin by training the model on the CNCC training set (the source domain) and we then retrain the model on either the keratocyte dataset or the keratinocyte dataset (the target domains). The transfer learning method involves initializing the network weights as the optimal weights for the source domain before retraining all weights on the target domain. This method relies on the assumption that our time series forecasting task shares some similarities, despite the differences in galvanotaxis dynamics between different cell types. Thus, the weight configuration of the source domain model is assumed to be a better starting point for the learning process on the target domain data than a random initialization, allowing for the network to converge to an accurate predictive model in less time and with fewer training instances.

### Generation of synthetic data

Because our model uses directedness and EF strength as input and predicts directedness, we can combine predicted directedness values with our choice of EF strength values to create synthetic input data which can in turn be fed back into the model to produce more directedness values. By using some initial lookback of ground truth experimental data to begin making predictions and some timeseries of EF values to combine with the predicted directedness values, we use our predictive model to simulate cell migration experiments of any length we wish. In this way, we can generate sequences of synthetic tracking data in which EF values are arbitrary and may vary in time.

### Plotting trajectories using simulations

To plot trajectories in the (x, y) plane and create rose plots using our synthetic data, we must map the directedness values generated by the model to (x, y) positions. These positions cannot be recovered exactly from the directedness values alone, so we approximate them using previous positions, as assumption of cell speed, and the previous heading of the cell.

We have the two previous positions, (x_i−1_, y_i−1_) and (x_i−2_, y_i−2_), and the next directedness value, cos (θ_i_), and we wish to approximate the next position, (x_i_, y_i_). We take the final speed value of the ground truth lookback, *s*, and take that to be the speed between (x_i−1_, y_i−1_) and (x_i_, y_i_), *s*_*i*_, because the speed of migrating CNCC tends to fluctuate more early in the experiment than later in the experiment, so we assume that speed will not fluctuate too much past the end of the ground truth lookback. Because there are two lines which pass through the origin and have directedness cos (θ_i_) and there are at most two points on each of those lines that can be reached by a cell starting at (x_i−1_, y_i–1_) and traveling at exactly speed *s*_*i*_, there are at most four candidate points for (x_i_, y_i_), (x_i_, y_i_)_j_ for *j* = 1,…,4.

To choose one of these candidate points, we consider the “heading” of the cell between (x_i−2_, y_i−2_) and (x_i−1_, y_i−1_). We define the heading as the angle of (x_i−1_, y_i−1_) − (x_i−2_,y_i−2_) relative to the electric field. While cells can change direction while migrating, it is not particularly common for direction to change drastically in a single timestep. Thus, we compute the heading between (x_i_, y_i_) and (x_i_, y_i_)_j_ for each *j* and take the candidate point whose heading is most similar to the heading between (x_i−2_, y_i−2_) and (x_i−1_, y_i−1_). That is, we take the point which assumes the least change in heading from that of the previous timestep. After choosing (x_i_, y_i_), we continue with the same process for (x_i+1_, y_i+1_).

### Feedback control simulations

For the simulation results seen in Fig. 9b, the PID parameters are as follows: Kp = 0.02, Ki = 0.002 and Kd = 0.008. The sampling size is set to 5 min to match the data.

## Supplementary Information

Supplementary Information.

## Data Availability

The machine learning models and datasets analysed during the current study are available in the GitHub repository, https://github.com/Gomez-Lab/galvanotaxis_cells.
