# Supplementary material for: A machine learning based model accurately predicts cellular response to electric fields in multiple cell types
Source: Sci Rep. 2022 Jun 15;12:9912. doi: 10.1038/s41598-022-13925-4 (PMC9200721; doi:10.1038/s41598-022-13925-4)
Supplement: Supplementary file 1 — Supplementary Information. [file 41598_2022_13925_MOESM1_ESM.docx]

**Supplementary materials for** A machine learning based model accurately predicts cellular response to electric fields in multiple cell types

**Authors:**

Brett Sargent^1^, Mohammad Jafari^2^, Giovanny Marquez^1^, Abijeet Singh Mehta^3,4^, Yao-Hui Sun^3,4^, Hsin-ya Yang^3^, Kan Zhu^3,4^, Roslyn Rivkah Isseroff^3^, Min Zhao^3,4^, Marcella Gomez^1*^

**Affiliations:**

^1^Department of Applied Mathematics, University of California, Santa Cruz, CA, USA.

^2^Department of Earth and Space Sciences, Columbus State University, Columbus, GA, USA.

^3^Department of Dermatology, University of California, Davis, Sacramento, CA, USA.

^4^Department of Ophthalmology & Vision Science, University of California, Davis, Sacramento, CA, USA.

**^*^Corresponding author/s:** Marcella Gomez (mgomez26@ucsc.edu)

Table S1: Sample of dataset.

| **EF Strength (V/mm)** | **Cell Number** | **Timestep** | **Directedness (cos θ)** |
| --- | --- | --- | --- |
| 0 | 1 | 1 | 0.000 |
| 0 | 1 | 2 | 0.990 |
| 0 | 1 | 3 | 0.986 |

Table S2: Median RMSE values and interquartile range (IQR) of RMSE distributions when predicting with all 50 models.

| **Model** | **Evaluation Set** | **Median RMSE** | **IQR of RMSE distribution** |
| --- | --- | --- | --- |
| Base | Train | 0.0311 | 0.0329 |
| Base | Validation | 0.0320 | 0.0382 |
| Base | Test | 0.0292 | 0.0345 |

Table S3: Average median RMSE across 5-fold CV for each normalization scheme.

| **EF Standardized** | **Directedness Standardized** | **Train Median RMSE** | **Validation Median RMSE** | **Test Median RMSE** |
| --- | --- | --- | --- | --- |
| False | False | 0.0289 | 0.0330 | 0.0280 |
| False | True | 0.0521 | 0.0592 | 0.0498 |
| True | False | 0.0295 | 0.0341 | 0.0291 |
| True | True | 0.0470 | 0.0658 | 0.0530 |

Table S4: Median RMSE values and interquartile range (IQR) of RMSE distributions

| **Model** | **Median RMSE** | **IQR of RMSE distribution** |
| --- | --- | --- |
| Constant Directedness model | .1687 | 0.0792 |
| Linear Predictor | 0.1975 | 0.1215 |

Table S5: Median RMSE values, interquartile range (IQR) of RMSE distributions, and performance relative to base model.

| **Model** | **Omitted EF** | **Test Set** | **Median RMSE** | **% difference in median over Base** | **IQR of RMSE distribution** |
| --- | --- | --- | --- | --- | --- |
| Base | N/A | Full | 0.0292 | N/A | 0.0345 |
| Base | N/A | 30mV/mm | 0.0340 | N/A | 0.0436 |
| Base | N/A | 200mV/mm | 0.0184 | N/A | 0.0162 |
| Interpolation | 30mV/mm | Full | 0.0308 | +5.48% | 0.0359 |
| Interpolation | 30mV/mm | 30mV/mm | 0.0354 | +4.12% | 0.0454 |
| Extrapolation | 200mV/mm | Full | 0.0311 | +6.61% | 0.0354 |
| Extrapolation | 200mV/mm | 200mV/mm | 0.0216 | +17.39% | 0.0262 |

Table S6: Median RMSE values for datasets used in transfer learning and performance relative to benchmark.

| **Prediction Task** | **Transfer Learning** | **Median RMSE** | **% difference in median over benchmark** | **% change in median from using transfer learning** | **IQR of RMSE distribution** |
| --- | --- | --- | --- | --- | --- |
| Polarity Reversal | No | 0.0459 | +57.1918% | N/A | 0.0250 |
| Polarity Reversal | Yes | 0.0376 | +28.7671% | -18.0828% | 0.0244 |
| Keratocyte | No | 0.0554 | +189.7260% | N/A | 0.0618 |
| Keratocyte | Yes | 0.0260 | -10.9589% | -53.0686% | 0.0268 |
| Keratinocyte 1 | No | 0.1010 | +244.5205% | N/A | 0.02502 |
| Keratinocyte 1 | Yes | 0.0362 | +23.9726% | -64.0159% | 0.0274 |
| Keratinocyte 2 | No | 0.2167 | +645.2055% | N/A | 0.3649 |
| Keratinocyte 2 | Yes | 0.1134 | +288.3562% | -47.8860% | 0.1522 |

Table S7: Means and medians of final directedness values for both ground truth and synthetic data. For simulations, these distributions are over 50 trained models to ensure that these results are not dependent on the random initialization of any one model; see Methods subsection Recurrent Model Architecture for more details.

|  | **0mV/mm** | **15mV/mm** | **30mV/mm** | **50mV/mm** | **75mV/mm** | **100mV/mm** | **200mV/mm** |
| --- | --- | --- | --- | --- | --- | --- | --- |
| **Real Mean** | -0.0071 | -0.1114 | -0.3266 | -0.4520 | -0.5210 | -0.8416 | -0.8718 |
| **Simulation Mean** | -0.1455 | -0.2531 | -0.3462 | -0.4333 | -0.5617 | -0.8260 | -0.8683 |
| **Real Median** | -0.1138 | -0.0852 | -0.5113 | -0.7071 | -0.7606 | -0.9349 | -0.9465 |
| **Simulation Median** | -0.2381 | -0.3920 | -0.5317 | -0.7298 | -0.8511 | -0.9531 | -0.9463 |


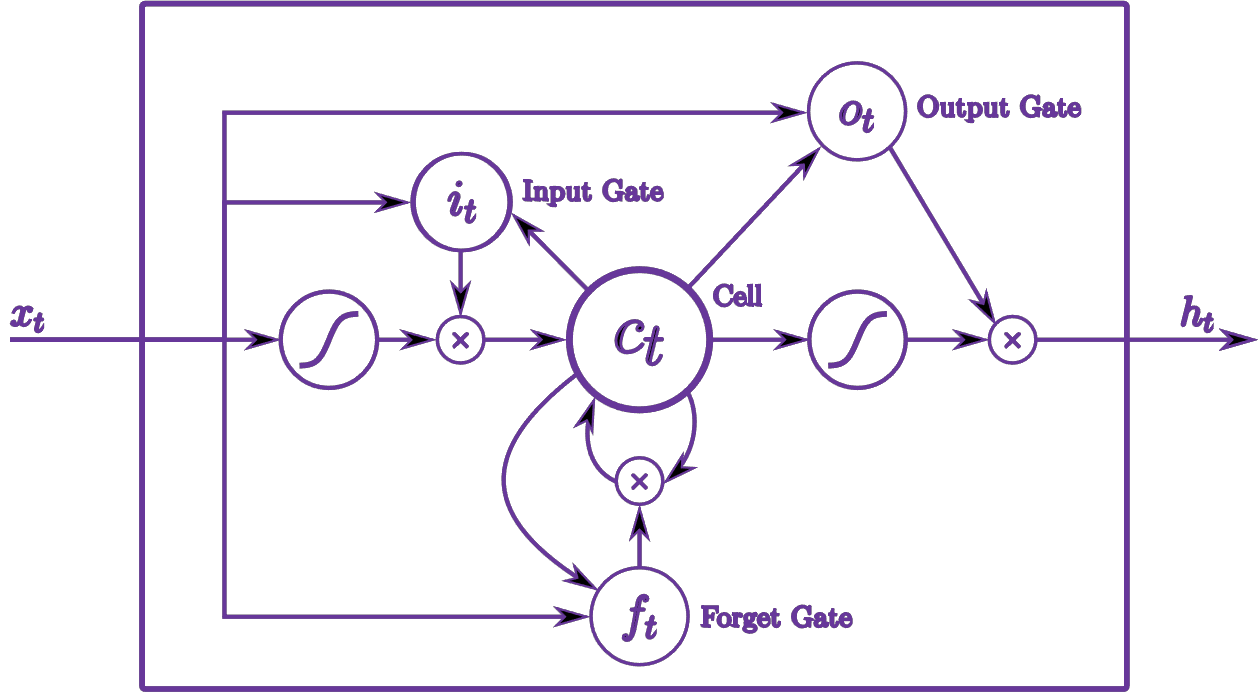


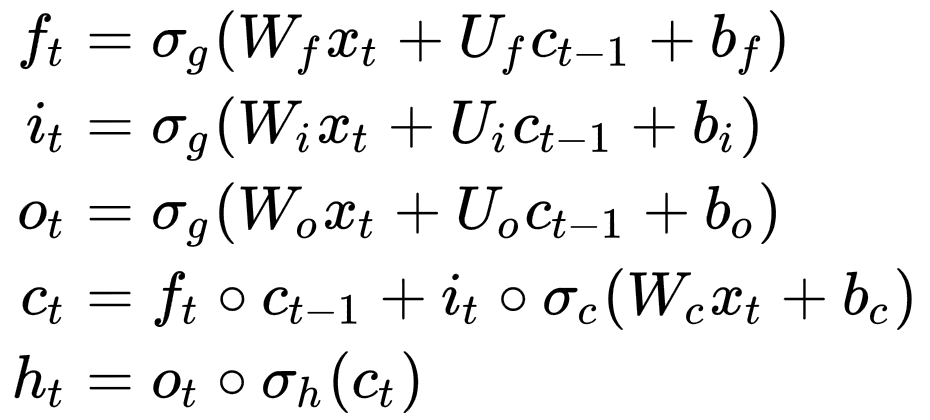


Supplementary Figure 1: A Long Short-Term Memory (LSTM) unit with input (it), output (ot), and forget (ft) gates. Each of these gates can be considered as a neuron in a feedforward neural network. W, 2U, and b are weight matrices and bias vector parameters which need to be learned during training. The operator “o” denotes the Hadamard product (element-wise product).

Supplementary Figure 1 shows a recurrent network with a gradient-based learning algorithm for updating its parameters. LSTM has feedback connections and can process entire sequences of time-series data. Since there can be delays of unknown duration between important events in a time-series, LSTM networks are advantages over other recurrent networks since they are relatively insensitive to the duration of said delays.


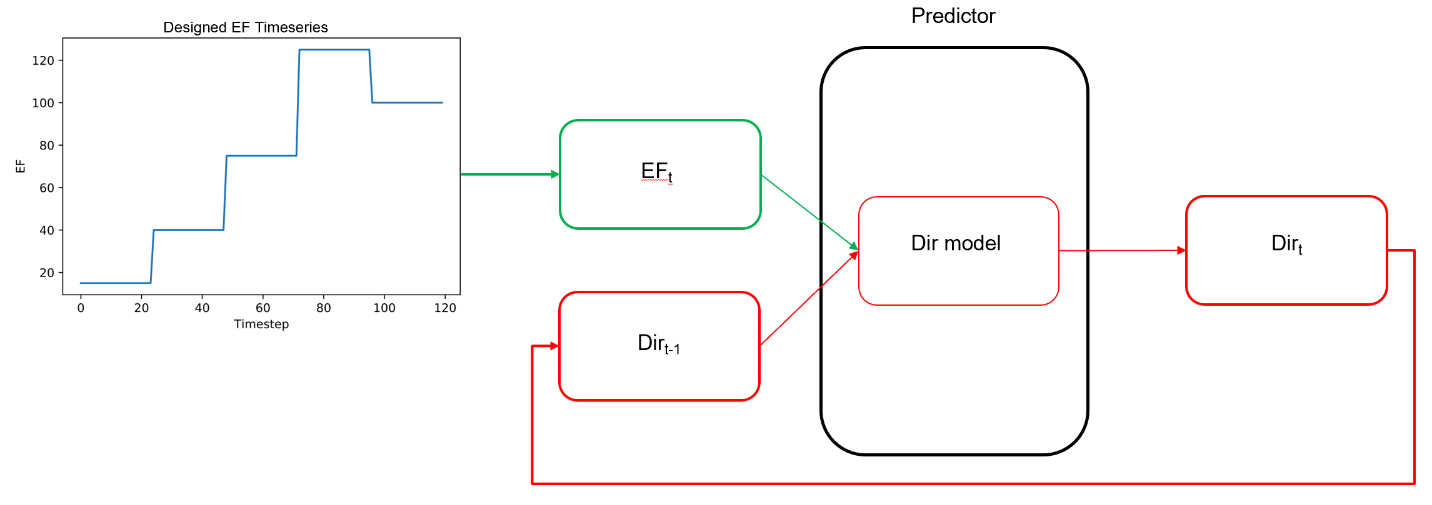


Figure S 2: Figure demonstrating offline simulation. Predicted values are fed back into the model instead of experimental ground truth data from a single cell.
